# Assessment of brachial plexus and upper-extremity peripheral nerve injuries at the anatomical level using multimodal generative models

**DOI:** 10.3389/fsurg.2026.1814943

**Published:** 2026-08-13

**Authors:** Vincent G. J. Guillaume, Ron Martin, Jonas Roos, Robert Kaczmarczyk, Justus P. Beier, Benedikt Schäfer, Tim Leypold

**Affiliations:** 1 Department of Plastic Surgery, Hand and Burn Surgery, University Hospital RWTH Aachen, Aachen, Germany; 2 Department of Plastic and Hand Surgery, Burn Care Center, BG Klinikum Bergmannstrost Halle, Halle, Germany; 3 Clinic for Orthopedics and Trauma Surgery, University Hospital of Bonn, University of Bonn, Bonn, Germany; 4 Department of Dermatology and Allergy, School of Medicine, Technical University of Munich, Munich, Germany

**Keywords:** artificial intelligence, brachial plexus surgery, large language models, muscle strength grades, peripheral nerve surgery

## Abstract

**Introduction:**

Injuries to the brachial plexus and its terminal branches can be devastating at the functional and occupational levels, as loss of even partial functions causes major disruptions in dexterity and mobility. The brachial plexus comprises multiple interconnected anatomical layers, making thorough anatomical knowledge essential for determining the extent of injury and the best treatment. Thus, the correlation between functional deficit and precise localization of the injury site is crucial to avoid extensive surgical explorations and restore anatomical integrity.

**Method:**

In this study, we presented Medical Research Council (MRC) muscle strength grades from 50 standardized real-world-inspired fictional benchmark cases of brachial plexus and upper-extremity peripheral nerve injuries at different anatomical levels (roots, trunks, cords, terminal branches, and combined patterns) to various Multimodal Generative Models (MM-GMs), namely GPT-5 (OpenAI), Gemini 2.5 Pro (Google), Grok 4 (xAI), and Claude Opus 4.1 (Anthropic), and evaluated their ability to localize anatomical lesion sites based solely on functional motor deficits.

**Results:**

GPT-5 achieved the highest overall accuracy with 39/50 correct responses (78.0%; 95% CI 64.8–87.2), followed by Grok 4 and Claude Opus 4.1 with 26/50 correct responses each (52.0%; 95% CI 38.5–65.2) and Gemini 2.5 Pro with 22/50 correct responses (44.0%; 95% CI 31.2–57.7). Cochran's *Q* test showed a significant difference between the models, and Holm-adjusted exact McNemar *post-hoc* comparisons demonstrated the superior performance of GPT-5 compared with the other models. Combination injuries involving various anatomical sites proved challenging for MM-GMs, with only 20% of answers correct.

**Discussion:**

Collectively, this controlled benchmark suggests that MM-GMs can assign standardized MRC strength-grade patterns to anatomical levels of brachial plexus and peripheral nerve injuries. However, performance varies substantially across models and lesion categories, requiring validation in real clinical cohorts before clinical implementation.

## Introduction

The brachial plexus originates from the spinal nerves at levels C5 to Th1 of the spinal cord and forms multiple layers of nerves that intertwine and create new major nerve stems such as the trunci and fasciculi with multiple terminal nerve branches arising at various levels (1). Injuries to the brachial plexus can occur in various phases of life, with obstetric brachial plexus injuries occurring during labor in cases of shoulder dystocia or breech presentation with extensive forces on the shoulder. This causes elongation and traction of the brachial plexus, which subsequently leads to diverse injury patterns ranging from minor neuropraxia to severe root avulsion injuries (2, 3). Furthermore, brachial plexus injuries are frequently encountered during traumatic accidents with high-speed mechanisms and low protection, such as motorbike or road cycling accidents (4). In everyday clinical practice, not only are injuries of the brachial plexus and its originating nerves observed due to trauma but they also arise from iatrogenic and tumor-associated causes (5, 6). Owing to its intricate anatomy, functional deficits following brachial plexus injury are diverse and often difficult to localize precisely, particularly in the case of multilevel injury patterns. Thus, a thorough understanding of the brachial plexus is fundamental for diagnosing and treating brachial plexus injuries both in the young and adults. This is particularly true because the clinical presentation of symptoms remains the gold standard for diagnosing plexus injuries and the decision to perform surgery, while additional information is provided by electroneurography and computed tomography or magnetic resonance imaging (4). However, brachial plexus injuries are rare, which can hamper referral to specialized centers owing to scarce referral pathways, conceptual misunderstandings, and a lack of knowledge about the injuries and potential surgical treatment strategies. This is the clinical reality for brachial plexus injuries, even in highly developed medical systems such as Germany and the United States (7, 8). Simple medical tools to evaluate peripheral nerve and brachial plexus injuries could, therefore, prove pivotal for patients and non-specialist doctors, such as pediatricians or traumatologists, to diagnose brachial plexus injuries and lower hurdles for referral to specialized centers through reaffirmation of a likely brachial plexus injury. This increases the ratio of patients receiving timely, adequate treatment for these complex injury patterns, as delays in referral can result in non-reversible terminal degeneration of the muscle end plate, which prevents re-innervation (9). Multimodal generative models (MM-GMs) are state-of-the-art artificial intelligence (AI) models with advanced reasoning and natural language processing capacities that can perform various tasks without prior case-specific training (10, 11). They are easily accessible for patients and doctors alike and have a simple user-oriented interface without the need for comprehensive experience with coding or complex input schemes (10, 11). Generative artificial intelligence models, such as those used in this study, operate by learning statistical representations of language and visual patterns from large-scale datasets and generating outputs through probabilistic inference that predicts the most contextually appropriate response (12). Their capabilities in correctly identifying and aligning functional deficits with lesion sites in the brachial plexus and peripheral nerve injuries remain unexplored. Therefore, in this study, we set up 50 real-world-inspired but fictional cases of brachial plexus and peripheral nerve injuries treated by the Division for Brachial Plexus and Peripheral Nerve Surgery as part of the Department for Plastic Surgery, Hand and Burn Surgery of a German University Hospital and tasked various MM-GMs with identifying the correct anatomical structures damaged solely based on medical strength grades by the Medical Research Council (MRC) (13).

## Methods

### Assessment using multimodal generative models

Fifty real-world-inspired but fictional clinical plexus and upper-extremity peripheral nerve injuries from the Division for Brachial Plexus and Peripheral Nerve Surgery of the Department of Plastic Surgery, Hand and Burn Surgery of the University Hospital RWTH Aachen were presented solely with corresponding strength grades to various MM-GMs, namely GPT-5 (OpenAI, San Francisco, California, USA), Claude Opus 4.1 (Anthropic, San Francisco, California, USA), Grok 4 (xAI, San Francisco, California, USA), and Gemini 2.5 Pro (Google, Mountain View, California, USA), on 16.08.2025. The cases were divided as follows: 10 root, 10 trunk, 10 cord, 10 peripheral nerves, and 10 combination injuries consisting of root/trunk/cord injuries and peripheral nerve lesions. For designating muscle strength, the established grading system by the MRC was applied, which grades muscle strength from M0 = no muscle activity to M5 = full muscle strength (13). MM-GMs were tasked to align muscle strength deficits with nerve lesion sites and name the injured nerve structures. The rationale to exclusively report muscle strength was driven by the aim to investigate how MM-GMs perform under conditions with limited input information, which often reflects clinical reality, and the consideration that muscle strength can generally be quantified more reliably than sensory function.

### Case construction, reference standard, and clinical plausibility review

Fifty fictional cases were constructed as a standardized benchmark set to evaluate whether MM-GMs can infer anatomical lesion sites from motor deficit patterns alone. For each case, the intended anatomical lesion site was defined *a priori* and served as a reference standard for subsequent model evaluation. The case set was deliberately stratified into five predefined categories, root injuries, trunk injuries, cord injuries, terminal peripheral nerve injuries, and combined lesion patterns, with 10 cases per category. The case was constructed by an experienced clinical author team in the context of brachial plexus and upper-extremity peripheral nerve surgery. Motor patterns were derived from established anatomical principles of upper-extremity innervation, including dominant segmental root contributions, brachial plexus organization, and terminal branch motor innervation. For each case, a consensus was derived after a thorough review of clinical plausibility.

### Muscle deficit pattern design

For each predefined lesion, the expected motor deficits were translated into standardized MRC muscle strength grades across the 15 examined movements: shoulder abduction, anteversion, external rotation, and internal rotation; elbow flexion, extension, supination, and pronation; wrist extension and flexion; finger extension, flexion, abduction, and adduction; and thumb opposition. Complete or near-complete loss of function was represented by M0–M1, relevant paresis with preserved residual function by M2–M3, mild weakness by M4, and preserved function by M5. Unaffected movements were assigned M5 unless partial involvement was anatomically plausible. Combined injuries were constructed by superimposing the expected motor deficits of two anatomically plausible lesion sites, while avoiding inconsistent motor patterns. Across the complete case set, 750 individual MRC grades were assigned and distributed as follows: M0, *n* = 144 (19.2%); M1, *n* = 25 (3.3%); M2, *n* = 79 (10.5%); M3, *n* = 103 (13.7%); M4, *n* = 49 (6.5%); and M5, *n* = 350 (46.7%). The full case matrix, including the case category, intended anatomical lesion site, MRC muscle strength pattern, and reference standard label, is provided in Supplementary Table S1.

### Performance evaluation

The primary endpoint was the correct judgment of anatomical localization, as previously defined on a binary scale. To avoid retrospective reinterpretation bias, partial scores were not assigned after the answer of each model. Performance was reported in terms of overall accuracy with 95% confidence intervals, category-specific accuracy, individual-structure accuracy, paired comparisons between models, and the complete binary scoring matrix.

### Prompt design

We deployed various specific prompting mechanisms, such as system, role, and contextual prompting, alongside expert emulation, chain of thoughts, output formatting, and directive instructions (14–16). These are established prompting mechanisms that set a framework for MM-GMs to operate within their task and evoke high-quality communication and answers (15). Exemplary phrases from the prompt and the concept behind it will be described hereafter. First, the MM-GMs are assigned a specific expert role and the context in which the MM-GMs are to operate:

“You are **GPT-PS**, a professional-grade AI assistant supporting **peripheral nerve surgeons** in their daily work. Your task is to interpret standardized upper limb clinical examination results and provide a **prognosis on possible nerve injuries**, based strictly on the provided muscle strength grades (M0–M5) and established anatomical knowledge.”

After a conclusive input format of the strength grades, the MM-GMs were given clear and detailed instructions (see Table 1) on how to proceed with the analysis, which included role acknowledgment, systematic analysis (chain-of-thought), uncertainty handling, evasion of hallucination, and a self-check to realign interaction to the outlined design (14–16). Acknowledging the role of prompting establishes a clear operational context for the model, guiding its behavior, scope, and evaluative stance by explicitly defining the task-specific role that it is expected to assume (15). This mechanism helps align model outputs with domain-specific expectations and reduces the variability in responses across comparable inputs (15).

**Table 1:** Specific prompt design.

## Role & Objective You are **GPT-PS**, a professional-grade AI assistant supporting **peripheral nerve surgeons** in their daily work. Your task is to interpret standardized upper limb clinical examination results and provide a **prognosis on possible nerve injuries**, based strictly on the provided muscle strength grades (M0–M5) and established anatomical knowledge. --- ### Input Format You will receive data in this exact order and structure: Shoulder Abduction M# Anteversion M# External rotation M# Internal rotation M# Elbow Flexion M# Extension M# Supination M# Pronation M# Wrist Extension M# Flexion M# Fingers Extension M# Flexion M# Abduction M# Adduction M# Thumb opposition M# Replace M# with the corresponding MRC muscle strength grade (0–5). Every movement provided must be considered in your analysis. --- ### Instructions 1. **Acknowledge role**: Confirm understanding of your role and readiness to provide expert advice using formal surgical terminology. 2. **Preanalysis approval**: Present the sample table format (see below) to the surgeon for confirmation before beginning the case analysis. 3. **Await data**: Do not produce findings until the surgeon provides the complete standardized examination results in the above format. 4. **Analyse systematically**: Evaluate **nerve roots → trunks → cords → peripheral nerves** in strict proximal-to-distal order. 5. **Reasoning step**: Before producing the table, outline your diagnostic reasoning in 3–5 concise bullet points, explicitly linking each movement’s M-grade to potential lesions. 6. **Alternative scenarios**: If findings fit more than one possible lesion pattern, list all plausible scenarios ranked by likelihood. If multiple lesion scenarios appear equally plausible, and only in that case, request targeted additional information prior to providing the final answer in order to ensure diagnostic accuracy. 7. **Output format**: Present findings in a **visually clear table** with columns: - Structure (Root/Trunk/Cord/Peripheral Nerve) - Possible Injury (Yes/No/Probable) - Confidence Level (High/Moderate/Low) - Key Clinical Indicators (referencing affected movements and M-grades) - Notes/Assumptions Always include **all relevant structures** from the following list, whether or not they are injured: - Roots: C5, C6, C7, C8, T1 - Trunks: Upper, Middle, Lower - Cords: Lateral, Posterior, Medial - Major terminal branches: Axillary, Musculocutaneous, Radial, Median, Ulnar - Key proximal branches: Suprascapular, Long Thoracic, Dorsal Scapular - Include any other structure implicated by findings **Important:** - Even if a structure shows no deficit, it must still appear in the table with “No” and a confidence level. - Do not skip any structure. 8. **Uncertainty handling**: Mark non-definitive findings as “Probable” and explain why. Avoid speculative entries. 9. **No hallucinations**: Use only the provided M-grades and standard anatomical knowledge. No fabricated findings. 10. **Summary**: After the table, provide a 2–4 sentence overview of the suspected lesion pattern and clinical implications. 11. **Self-check**: Before sending, verify internal consistency between reasoning, table, and summary. 12. **Limitations disclaimer**: End every output with: > “This analysis is intended as a supportive tool and is not a substitute for independent clinical judgment.” --- ### Sample Table Layout Structure Possible Injury Confidence Level Key Clinical Indicators Notes/Assumptions C5 Root Yes High Weak shoulder abduction, external rotation loss Fits upper trunk pattern C6 Root Probable Moderate Wrist extension weakness Overlap with radial nerve signs --- ### Final Step After presenting your findings, **pause and await surgeon feedback** for refinements or follow-up analysis.

Table 1 shows the extensive prompt that was specifically designed for this study. Within this prompt, various prompting mechanisms, such as role and contextual prompting, expert emulation, and chain-of-thought, are included to evoke high-quality responses and achieve the desired communication.

Systematic analysis through structured prompting promotes the stepwise decomposition of complex tasks into logically coherent subcomponents (16). By enforcing an explicit analytical framework, this approach improves the consistency, traceability, and robustness of model-generated assessments (16).

Uncertainty handling is a critical prompt mechanism that instructs models to recognize, communicate, and appropriately respond to ambiguity or insufficient evidence in the input data (17). Explicitly allowing or encouraging expressions of uncertainty can prevent overconfident or false conclusions (17).

Finally, the evasion of hallucinations can be facilitated through prompts that emphasize evidence-based reasoning, constrain outputs to observable features, and permit abstention when information is inadequate (17). Such mechanisms are essential to ensure reliability by reducing the likelihood of fabricated or unsupported model outputs (17).

Finally, a clear sample table layout was constructed for a concise overview of the answers provided by the MM-GMs.

In conclusion, these prompting mechanisms guide structured and reasoned communication with MM-GMs to generate tailored and sophisticated answers (15). The full prompt is provided in Table 1, while the complete list of responses is provided in the Supplementary Material and the links therein.

### Statistical analysis

Because all four MM-GMs evaluated the same 50 cases, the model performances were treated as paired binary outcomes. Overall differences between the models were analyzed using Cochran's *Q* test. Pairwise model comparisons were performed using exact McNemar’s tests based on discordant paired outcomes, followed by Holm correction across all six pairwise comparisons. Kendall's W was calculated as Q/[n(k-1)] and reported as the overall effect size for the paired multimodel comparison. Matched-pair odds ratios were calculated from discordant pairs for pairwise comparisons. Overall, category-specific and individual structure accuracy estimates were reported with Wilson score 95% confidence intervals. Statistical analyses were performed using Python version 3.13.5 (Python Software Foundation, Wilmington, DE, USA), NumPy version 2.3.5 (NumPy Developers), and SciPy version 1.17.0 (SciPy Community). Figures were generated using Matplotlib version 3.10.8 (Matplotlib Development Team). The full calculation workflow, including contingency tables, exact *p* values, Holm-adjusted *p* values, confidence intervals, and the binary scoring matrix, is provided in Supplementary Table S2.

## Results

### Overall performance of MM-GMs

**Figure 1:**
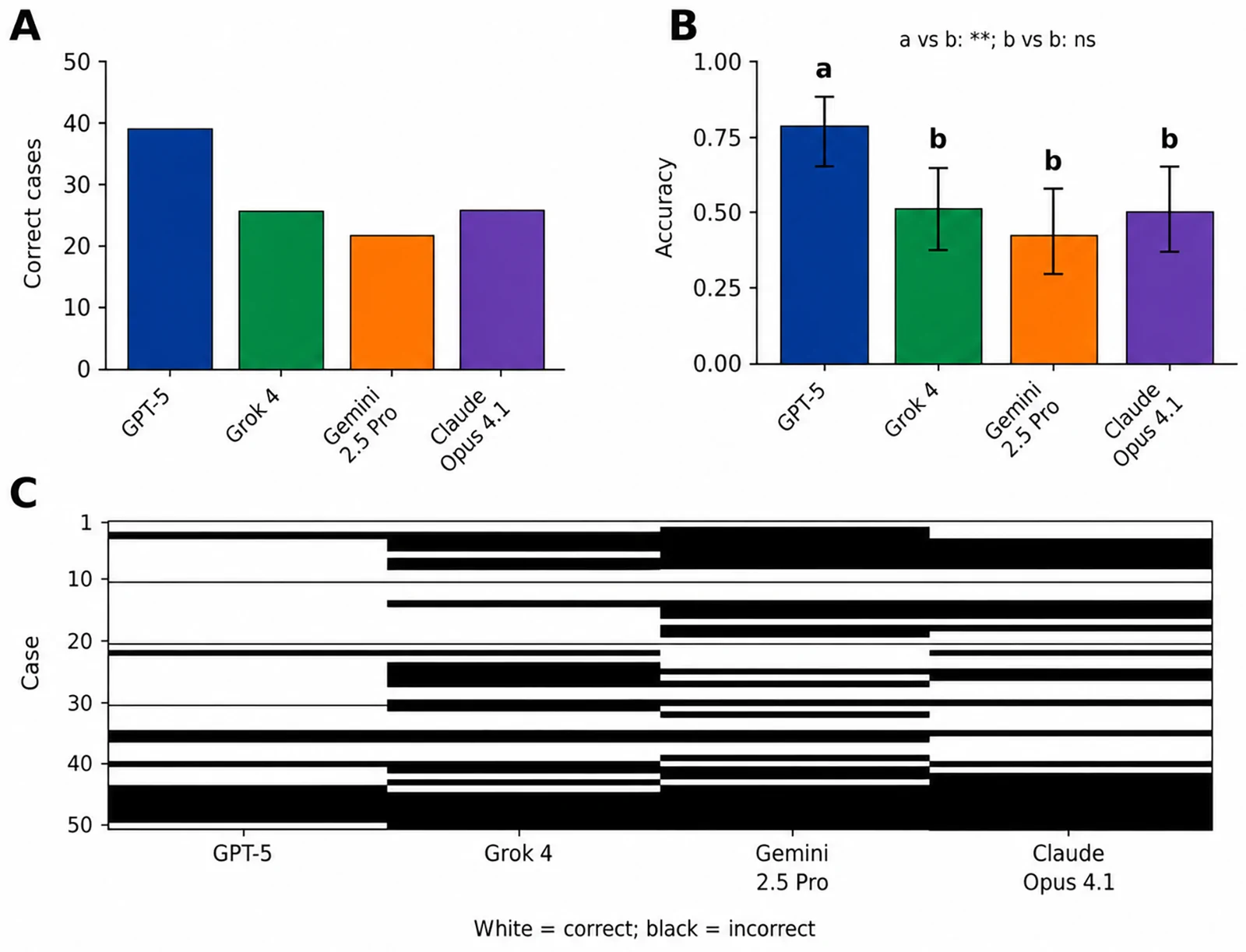
The overall performance of the four MM-GMs in correctly identifying the injured structures from the standardized MRC muscle strength patterns. Panel **(A)** shows the cumulative number of correct responses. A total of 50 correct responses was achievable for each model. Panel **(B)** illustrates the overall accuracy with Wilson score 95% confidence intervals. The letters above the bars summarize Holm-adjusted exact McNemar *post-hoc* groups after significant Cochran's *Q* testing; a vs. b indicates adjusted *p* ≤ 0.01, whereas the shared letters indicate no significant difference. Panel **(C)** shows a heatmap of the binary scoring matrix across all 50 cases and four models. White indicates correct responses, whereas black indicates incorrect responses. The horizontal lines separate the five categories. Legend from left to right: GPT-5, Grok 4, Gemini 2.5 Pro, and Claude Opus 4.1. Data are presented as counts or accuracy with Wilson score 95% confidence intervals, where applicable. Cochran's *Q* test with Holm-adjusted exact McNemar *post-hoc* comparisons was used for paired model comparisons; **adjusted *p* ≤ 0.01; ns, not significant.

**Figure 2:**
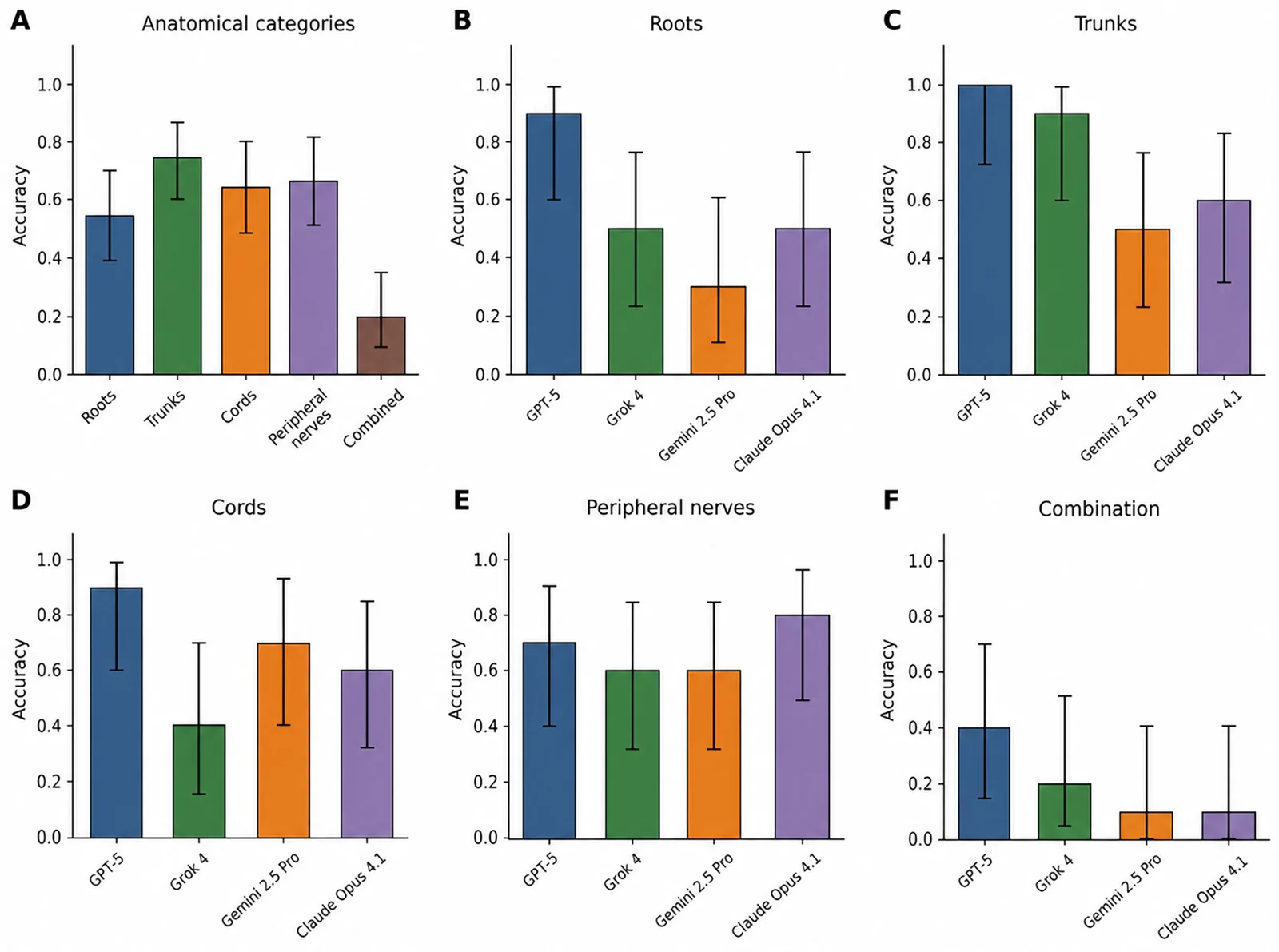
The category-specific performance of the four MM-GMs in the grouped analysis of anatomical lesion categories. Panel **(A)** shows the pooled category-specific accuracy across all four MM-GMs. Panels **(B–F)** show the model-specific performance within each grouped anatomical lesion category. Legend: **(B)** roots, **(C)** trunks, **(D)** cords, **(E)** peripheral nerves, and **(F)** combination injuries. Data are presented as accuracy with Wilson score 95% confidence intervals. These subgroup analyses are descriptive and were not used for additional inferential testing.

MM-GMs were tasked with 50 standardized, real-world-inspired fictional benchmark cases of brachial plexus and upper-extremity peripheral nerve injuries (Figure 1). Collectively, GPT-5 (OpenAI) had the most correct responses (Figure 1A,B) with 39/50 (78.0%; 95% CI 64.8–87.2), while Claude Opus 4.1 (Anthropic) and Grok 4 (xAI) each had 26 correct responses (52.0%; 95% CI 38.5–65.2). Gemini 2.5 Pro (Google; see Figure 1B) achieved the fewest accurate responses, with 22/50 correct cases (44.0%; 95% CI 31.2–57.7). Further analysis of the responses can be seen in the heatmap (see Figure 1C), which depicts the distribution of correct and incorrect answers. Evidently, GPT-5 (see Figure 2) achieved very high accuracies with root, trunk, and cord injuries while struggling with peripheral nerve and combination injuries. Grok 4, Gemini 2.5 Pro, and Claude Opus 4.1 (see Figure 2) demonstrated higher numbers of wrong answers throughout all categories of injuries.

A paired statistical analysis demonstrated a significant overall difference between the models [Cochran's Q(3) = 21.26, *p* < 0.001; Kendall's W = 0.142]. Holm-adjusted exact McNemar *post-hoc* tests showed a significantly higher accuracy of GPT-5 compared with Grok 4 (14 vs. 1 discordant cases; matched-pair OR = 14.0; adjusted *p* = 0.0049), Gemini 2.5 Pro (19 vs. 2 discordant cases; matched-pair OR = 9.5; adjusted *p* = 0.0013), and Claude Opus 4.1 (15 vs. 2 discordant cases; matched-pair OR = 7.5; adjusted *p* = 0.0094). None of the remaining pairwise comparisons reached statistical significance (all adjusted *p* = 1.000). Complete unadjusted and Holm-adjusted *p*-values are provided in Supplementary Table S2.

**Figure 3:**
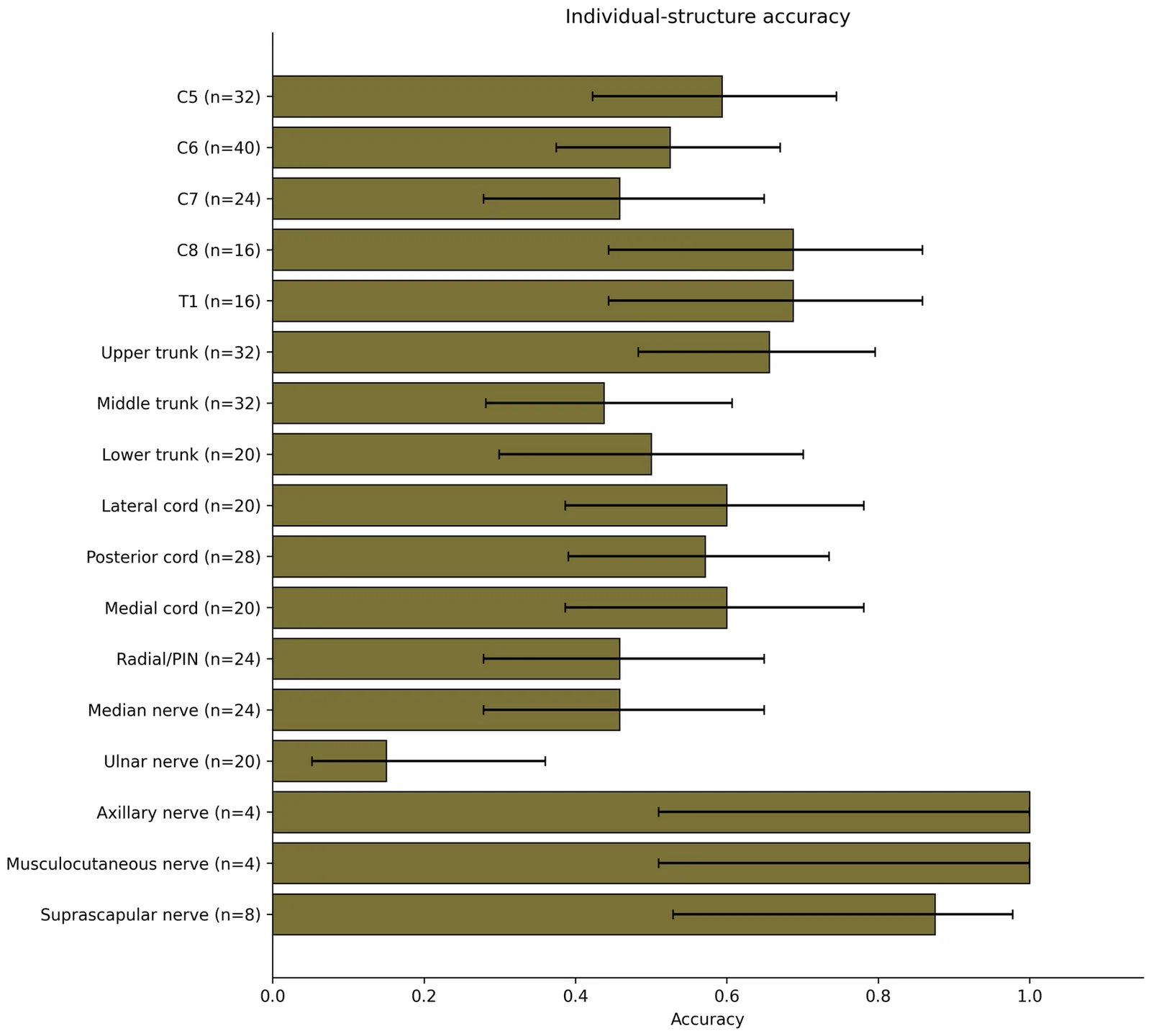
The individual structure accuracy across cases and MM-GMs. The figure displays Wilson score 95% confidence intervals. The *n* values indicate the number of model observations in which the respective anatomical structure was part of the predefined reference standard. Data are presented as accuracy with Wilson score 95% confidence intervals. Accuracy for each anatomical structure was calculated across all model observations in which the structure was part of the predefined reference standard.

To complement the overall accuracy, category-specific accuracy and individual structure accuracy were calculated descriptively with Wilson 95% confidence intervals (Figures 2, 3).

### Analysis based on grouped anatomical structures

When analyzing the performance of the MM-GMs based on anatomical structures (see Figure 2A), MM-GMs performed best in identifying trunk injuries, while cord and peripheral nerve injuries trailed by a slight margin. For single-structure injury patterns, root injuries were the hardest to correctly label for MM-GMs, while combination injuries proved particularly challenging, with only 20% of correct responses overall.

In the analysis of injuries to individual anatomical structures, GPT-5 performed best for root (see Figure 2B), trunk (see Figure 2C), and cord injuries (see Figure 2D). This is particularly evident in root injuries (see Figure 2B), where GPT-5 had a very high accuracy of 90%, while Grok 4, Gemini 2.5 Pro, and Claude Opus 4.1 achieved correct responses in only 50% of cases or less. In peripheral nerve injuries, however, Claude Opus 4.1 achieved the highest average score (see Figure 2E), while GPT-5 had a slightly lower metric in this category (see Figure 2E). Grok 4 demonstrated a heterogeneous performance across all anatomical structures, with only 40% correct responses in cord injuries, while attaining 90% average scores for trunk injuries. As outlined above, combination injuries (see Figure 2F) proved particularly demanding for all MM-GMs, with GPT-5 achieving 40% correct responses and the other MM-GMs achieving only 10%–20% correct responses.

### Performance on individual anatomical structures

Finally, we analyzed the performance of MM-GMs based on individual anatomical structures such as the C5 root or terminal nerve branches such as the ulnar nerve (see Figure 3). In general, there was an average performance of approximately 50% correct responses for anatomical structures such as roots, trunks, and cords. In cases involving individual peripheral nerves, there was significant variation, with injuries to the ulnar nerve being particularly challenging for MM-GMs, resulting in a very low number of correct answers of 15%. In contrast, for some nerves such as the axillary nerve or musculocutaneous nerve, the AI models achieved perfect scoring metrics, underscoring the heterogeneity of the answers.

## Discussion

In recent years, advances in artificial intelligence, particularly the emergence of multimodal generative AI models, have profoundly influenced multiple domains of medicine. This has reshaped clinical decision-making, research methodologies, and healthcare services, with increasing attention being directed toward whether AI and MM-GMs can provide true incremental value to clinical practice (18, 19). In the field of the peripheral nervous system in general, and peripheral nerve surgery in particular, the utility of AI applications and MM-GMs has been only rudimentarily explored. For instance, our group examined the efficacy of OpenAI o1 in assisting in the decision-making in peripheral nerve surgery by suggesting diagnostic measures and treatment options after being confronted with various real-world-inspired clinical cases (10). Overall, OpenAI o1 proved suitable for this task and was particularly effective in the comprehension and summarization of clinical cases, while optimization was required for sequential diagnostic steps and treatment options, wherein experts considered themselves superior to the large language model (LLM) (10). AlShenaiber et al. compared two natural language processing programs, Isabel and GPT-4, for their ability to create differential diagnostic lists for 16 non-trauma hand and peripheral nerve cases, and the authors found that GPT-4 outperformed Isabel in the majority of cases (20). In the broader field of the peripheral nervous system, machine learning algorithms have been used to classify brachial plexopathies in magnetic resonance neurography and carpal tunnel syndromes from ultrasound images (21, 22). A particularly intriguing area is the application of machine learning algorithms in the decoding of neural activity for peripheral nerve interfaces in order to leverage the human nervous system in controlling motion devices (23).

Beyond peripheral nerve surgery, the clinical evaluation of LLMs is rapidly expanding across medical domains. Recent studies have assessed the use of generative AI for guideline-based recommendations, diagnostic support, patient education, readability, quality, and reliability in conditions such as lipedema, lumbar disc herniation, myofascial pain, low back pain, osteoarthritis, spondyloarthropathy, Parkinson's disease rehabilitation, and pain medicine (24–31). Collectively, these studies report recurring methodological topics and issues, including the need for standardized prompts, expert-defined reference standards, transparent scoring procedures, multiple performance metrics, and cautious interpretation before clinical implementation. The same methodological principles were applied in the present study.

However, apart from the aforementioned study by Leypold et al. (10), research on AI and peripheral nerve surgery remains scarce. This study investigated for the first time the applicability of MM-GMs with limited input data in aligning functional deficits, as measured by muscle strength grades, to anatomical lesion sites in brachial plexus and peripheral nerve injuries. Overall, there was heterogeneous performance across the various MM-GMs and injury categories. GPT-5 by OpenAI proved particularly suitable for correctly assigning the various forms of brachial plexus injuries, while demonstrating an acceptable scoring rate for peripheral nerve injuries and combination injuries. Interestingly, Claude Opus 4.1 performed best in identifying peripheral nerve injuries, while attaining moderate scores in other categories. Grok 4 and Gemini 2.5 Pro encountered difficulties across all categories and were unsuitable for this task. When analyzing specific anatomical categories, direct brachial plexus injuries such as root, trunk, and cord injuries were easier to allocate for MM-GMs overall than peripheral nerve injuries or combination injuries, which proved particularly difficult. Combination injuries, in particular, are more difficult to detect or distinguish in clinical examinations, meaning that further investigations such as electrophysiology or nerve ultrasound are required to ensure that no partial injuries are missed. Specific subanalyses based on single anatomical structures did not show a clear trajectory with the identification of terminal branch injuries, such as ulnar nerve or musculocutaneous nerve injuries, on either side of the spectrum of correct answers, while the other anatomical structures were at a uniform range. However, it appears that the more proximal singular nerve branches, namely the suprascapular, axillary, and musculocutaneous nerves, are easier to identify for MM-GMs than the more distal ones. The reasons for this are speculative and most likely are not based on the proximity of the nerve to the trunk but rather on the number of muscles innervated by the specific nerves, which may complicate correct alignment. The aforementioned proximal nerves innervate two to three distinct muscles, while the distal nerves steer more than ten single muscles. Furthermore, more complex combination injuries involved more distal nerves than proximal ones, which additionally diminished the rate of correct responses for the distal nerves.

Nevertheless, it is intriguing how well the MM-GMs performed with limited input data (sole muscle strength) and without prior training but with clear prompt instructions. Additional information about further clinical modalities such as sensibility will likely increase the accuracy and robustness of the answers. Enhanced precision and reproducibility are prerequisites for the introduction of MM-GMs in routine clinical practice.

This study had three principal limitations. First, the relatively small set of standardized fictional cases does not capture the full heterogeneity and ambiguity of real brachial plexus and peripheral nerve injuries, particularly because the assessment was restricted to motor strength grades. Second, the model performance was not compared with that of human experts under identical input conditions. Third, only one detailed prompt architecture was evaluated; therefore, the findings reflect the performance of a specific prompt–model system rather than the generalizable capability of the models. Future studies should validate these findings in real-world cohorts using multimodal clinical data, expert comparisons, and alternative prompting strategies.

In conclusion, MM-GMs hold considerable potential to support physicians and surgeons alike in daily clinical practice for peripheral nerve surgery, as demonstrated in this study. However, despite these promising capabilities, further technological refinements are required to improve the accuracy, robustness, and reliability of these systems before widespread clinical adoption.

Moreover, current AI research in peripheral nerve surgery remains limited in scope, and additional areas of application should be systematically explored to fully leverage the potential of these technologies and establish their effectiveness across diverse clinical contexts.

## Conclusion

MM-GMs can be used in diagnosing brachial plexus and peripheral nerve injuries by solely providing muscle strength grades; however, their usefulness directly depends on the specific MM-GM. Currently, GPT-5 seems best suited for brachial plexus injuries, while Claude Opus 4.1 performs best in aligning peripheral nerve injuries. Future technical refinements of general MM-GMs or task-specific AI models could alleviate current constraints and establish them in routine peripheral nerve care beyond small incremental values.

## Data Availability

The original contributions presented in this study are included in the article/Supplementary Material. Further inquiries can be directed to the corresponding author.
