# Supplementary material for: Assessment of brachial plexus and upper-extremity peripheral nerve injuries at the anatomical level using multimodal generative models
Source: Front Surg. 2026 Aug 13;13:1814943. doi: 10.3389/fsurg.2026.1814943 (PMC13518590; doi:10.3389/fsurg.2026.1814943)
Supplement: Supplementary file 1 [file Datasheet1.docx]

**Supplementary Table 1. Complete fictional case matrix and reference-standard lesion labels**

The table lists the 50 standardized real-world-inspired fictional benchmark cases used for MM-GM testing. Each case contains the predefined reference-standard lesion label and the corresponding MRC motor grades for the 15 standardized movements.

**A. Reference-standard lesion labels**

| **Case** | **Category** | **Reference-standard anatomical lesion label** |
| --- | --- | --- |
| 1 | Root | Lesion of the nerve roots C5–C6, complete |
| 2 | Root | Incomplete C5 + C6 lesion |
| 3 | Root | Complete lesion C5 + C6 + C7 |
| 4 | Root | Incomplete C5 + C6 + C7 lesion |
| 5 | Root | Complete lesion C8 + T1 |
| 6 | Root | Incomplete C8 + T1 lesion |
| 7 | Root | Radicular C6 lesion (foraminal fracture) |
| 8 | Root | Dominant C7 avulsion with involvement of C6 |
| 9 | Root | Global avulsion C5–T1 (complete) |
| 10 | Root | Global but incomplete C5–T1 contusion |
| 11 | Trunk | Complete superior trunk lesion |
| 12 | Trunk | Partial superior trunk lesion |
| 13 | Trunk | Mild contusion of the superior trunk |
| 14 | Trunk | Complete lesion of the superior + middle trunks |
| 15 | Trunk | Incomplete lesion of the superior + middle trunks |
| 16 | Trunk | Complete middle trunk lesion |
| 17 | Trunk | Partial middle trunk lesion |
| 18 | Trunk | Complete inferior trunk lesion |
| 19 | Trunk | Partial inferior trunk lesion |
| 20 | Trunk | Incomplete global trunk contusion |
| 21 | Cord | Complete lesion of the lateral cord |
| 22 | Cord | Partial lesion of the lateral cord |
| 23 | Cord | Complete lesion of the posterior cord |
| 24 | Cord | Partial lesion of the posterior cord |
| 25 | Cord | Complete lesion of the medial cord |
| 26 | Cord | Partial lesion of the medial cord |
| 27 | Cord | Complete lesions of the lateral + posterior cords |
| 28 | Cord | Complete lesions of the posterior + medial cords |
| 29 | Cord | Complete lesions of the lateral + medial cords |
| 30 | Cord | Global lesion of all three cords with intact roots and trunks |
| 31 | Peripheral nerve | Radial nerve lesion (distal to the triceps branch) |
| 32 | Peripheral nerve | Posterior interosseous nerve lesion |
| 33 | Peripheral nerve | High median nerve lesion (proximal to the pronator teres) |
| 34 | Peripheral nerve | Distal median nerve lesion (carpal tunnel) |
| 35 | Peripheral nerve | High ulnar nerve lesion (cubital tunnel) |
| 36 | Peripheral nerve | Distal ulnar nerve lesion (Guyon’s canal) |
| 37 | Peripheral nerve | Musculocutaneous nerve lesion |
| 38 | Peripheral nerve | Axillary + Suprascapular nerve lesion |
| 39 | Peripheral nerve | Suprascapular nerve lesion |
| 40 | Peripheral nerve | Combined distal median and ulnar nerve lesion (wrist laceration) |
| 41 | Combination | C5–C6 avulsion + high radial nerve lesion (distal to the triceps branch) |
| 42 | Combination | Incomplete superior trunk lesion + high radial nerve lesion (distal to the triceps branch) |
| 43 | Combination | Complete posterior cord lesion + distal median nerve lesion (carpal tunnel) |
| 44 | Combination | Partial middle trunk lesion + high ulnar nerve lesion (cubital tunnel) |
| 45 | Combination | C7 radiculopathy + posterior interosseous nerve syndrome |
| 46 | Combination | Complete inferior trunk lesion + distal median nerve lesion (carpal tunnel) |
| 47 | Combination | Incomplete lesion of the superior + middle trunks + distal ulnar nerve lesion (Guyon’s canal) |
| 48 | Combination | C5–C6 avulsion + complete lesion of the posterior cord |
| 49 | Combination | Incomplete middle trunk lesion (C7) + high median nerve lesion (proximal to the pronator teres) |
| 50 | Combination | Partial inferior trunk lesion (C8–T1) + high radial nerve lesion (distal to the triceps branch) |

**B. MRC muscle strength-grade matrix**

| **Case** | **Sh abd** | **Sh ante** | **Sh ext rot** | **Sh int rot** | **Elb flex** | **Elb ext** | **Supination** | **Pronation** | **Wrist ext** | **Wrist flex** | **Finger ext** | **Finger flex** | **Finger abd** | **Finger add** | **Thumb opp** |
| --- | --- | --- | --- | --- | --- | --- | --- | --- | --- | --- | --- | --- | --- | --- | --- |
| 1 | M0 | M3 | M0 | M3 | M0 | M5 | M0 | M5 | M4 | M5 | M4 | M5 | M5 | M5 | M5 |
| 2 | M3 | M3 | M2 | M5 | M3 | M5 | M2 | M5 | M3 | M5 | M5 | M5 | M5 | M5 | M5 |
| 3 | M0 | M0 | M0 | M2 | M0 | M0 | M0 | M1 | M2 | M2 | M0 | M3 | M3 | M3 | M3 |
| 4 | M2 | M3 | M2 | M4 | M2 | M3 | M2 | M3 | M2 | M3 | M2 | M4 | M4 | M4 | M4 |
| 5 | M5 | M5 | M5 | M5 | M5 | M5 | M5 | M5 | M4 | M0 | M0 | M0 | M0 | M0 | M0 |
| 6 | M5 | M5 | M5 | M5 | M5 | M5 | M5 | M5 | M4 | M3 | M3 | M3 | M2 | M2 | M2 |
| 7 | M4 | M4 | M4 | M5 | M3 | M5 | M3 | M5 | M3 | M5 | M5 | M5 | M5 | M5 | M5 |
| 8 | M3 | M3 | M3 | M4 | M3 | M0 | M3 | M0 | M2 | M0 | M0 | M3 | M3 | M3 | M3 |
| 9 | M0 | M0 | M0 | M0 | M0 | M0 | M0 | M0 | M0 | M0 | M0 | M0 | M0 | M0 | M0 |
| 10 | M2 | M2 | M2 | M3 | M2 | M2 | M2 | M2 | M2 | M2 | M2 | M2 | M1 | M1 | M1 |
| 11 | M0 | M0 | M0 | M2 | M0 | M5 | M0 | M5 | M2 | M5 | M5 | M5 | M5 | M5 | M5 |
| 12 | M3 | M3 | M2 | M4 | M3 | M5 | M3 | M5 | M3 | M5 | M5 | M5 | M5 | M5 | M5 |
| 13 | M4 | M4 | M3 | M5 | M4 | M5 | M4 | M5 | M4 | M5 | M5 | M5 | M5 | M5 | M5 |
| 14 | M0 | M0 | M0 | M1 | M0 | M0 | M0 | M1 | M0 | M2 | M0 | M3 | M3 | M3 | M3 |
| 15 | M2 | M2 | M2 | M4 | M2 | M3 | M2 | M3 | M2 | M3 | M2 | M4 | M4 | M4 | M4 |
| 16 | M5 | M5 | M5 | M5 | M5 | M0 | M5 | M0 | M0 | M1 | M0 | M5 | M5 | M5 | M5 |
| 17 | M5 | M5 | M5 | M5 | M5 | M3 | M5 | M3 | M3 | M3 | M3 | M5 | M5 | M5 | M5 |
| 18 | M5 | M5 | M5 | M5 | M5 | M5 | M5 | M5 | M4 | M0 | M0 | M0 | M0 | M0 | M0 |
| 19 | M5 | M5 | M5 | M5 | M5 | M5 | M5 | M5 | M4 | M3 | M5 | M3 | M2 | M2 | M2 |
| 20 | M2 | M2 | M2 | M3 | M2 | M2 | M2 | M2 | M2 | M2 | M2 | M2 | M1 | M1 | M1 |
| 21 | M5 | M3 | M5 | M5 | M0 | M5 | M0 | M4 | M5 | M3 | M5 | M4 | M5 | M5 | M4 |
| 22 | M5 | M4 | M5 | M5 | M3 | M5 | M3 | M5 | M5 | M4 | M5 | M5 | M5 | M5 | M5 |
| 23 | M1 | M3 | M1 | M2 | M5 | M0 | M5 | M5 | M0 | M5 | M0 | M5 | M5 | M5 | M5 |
| 24 | M3 | M4 | M3 | M4 | M5 | M3 | M5 | M5 | M3 | M5 | M3 | M5 | M5 | M5 | M5 |
| 25 | M5 | M5 | M5 | M5 | M5 | M5 | M5 | M4 | M5 | M2 | M5 | M2 | M0 | M0 | M0 |
| 26 | M5 | M5 | M5 | M5 | M5 | M5 | M5 | M4 | M5 | M3 | M5 | M3 | M2 | M2 | M2 |
| 27 | M1 | M2 | M1 | M2 | M0 | M0 | M0 | M4 | M0 | M2 | M0 | M3 | M5 | M5 | M4 |
| 28 | M1 | M2 | M1 | M2 | M5 | M0 | M5 | M4 | M0 | M2 | M0 | M2 | M0 | M0 | M0 |
| 29 | M5 | M3 | M5 | M5 | M0 | M5 | M0 | M0 | M5 | M0 | M5 | M0 | M0 | M0 | M0 |
| 30 | M2 | M0 | M2 | M2 | M0 | M0 | M0 | M0 | M0 | M0 | M0 | M0 | M0 | M0 | M0 |
| 31 | M5 | M5 | M5 | M5 | M5 | M5 | M3 | M5 | M0 | M5 | M0 | M5 | M5 | M5 | M5 |
| 32 | M5 | M5 | M5 | M5 | M5 | M5 | M3 | M5 | M3 | M5 | M0 | M5 | M5 | M5 | M5 |
| 33 | M5 | M5 | M5 | M5 | M5 | M5 | M5 | M0 | M5 | M3 | M5 | M2 | M5 | M5 | M0 |
| 34 | M5 | M5 | M5 | M5 | M5 | M5 | M5 | M5 | M5 | M5 | M5 | M5 | M5 | M5 | M0 |
| 35 | M5 | M5 | M5 | M5 | M5 | M5 | M5 | M5 | M5 | M4 | M4 | M3 | M0 | M0 | M3 |
| 36 | M5 | M5 | M5 | M5 | M5 | M5 | M5 | M5 | M5 | M5 | M5 | M5 | M0 | M0 | M4 |
| 37 | M5 | M5 | M5 | M5 | M0 | M5 | M2 | M5 | M5 | M5 | M5 | M5 | M5 | M5 | M5 |
| 38 | M0 | M2 | M2 | M5 | M5 | M5 | M5 | M5 | M5 | M5 | M5 | M5 | M5 | M5 | M5 |
| 39 | M3 | M5 | M3 | M5 | M5 | M5 | M5 | M5 | M5 | M5 | M5 | M5 | M5 | M5 | M5 |
| 40 | M5 | M5 | M5 | M5 | M5 | M5 | M5 | M5 | M5 | M0 | M5 | M0 | M0 | M0 | M0 |
| 41 | M0 | M1 | M0 | M3 | M0 | M5 | M0 | M5 | M0 | M5 | M0 | M5 | M5 | M5 | M5 |
| 42 | M2 | M3 | M2 | M4 | M3 | M5 | M3 | M5 | M1 | M5 | M1 | M5 | M5 | M5 | M5 |
| 43 | M3 | M3 | M3 | M5 | M5 | M0 | M3 | M5 | M0 | M5 | M0 | M5 | M5 | M5 | M0 |
| 44 | M5 | M5 | M5 | M5 | M5 | M5 | M5 | M3 | M3 | M3 | M3 | M3 | M0 | M0 | M5 |
| 45 | M5 | M5 | M5 | M5 | M5 | M2 | M4 | M3 | M1 | M5 | M0 | M5 | M5 | M5 | M5 |
| 46 | M5 | M5 | M5 | M5 | M5 | M5 | M5 | M3 | M5 | M3 | M5 | M3 | M0 | M0 | M0 |
| 47 | M2 | M2 | M2 | M3 | M3 | M4 | M3 | M3 | M3 | M3 | M3 | M3 | M0 | M0 | M1 |
| 48 | M0 | M1 | M0 | M2 | M1 | M0 | M0 | M4 | M0 | M5 | M0 | M5 | M5 | M5 | M5 |
| 49 | M5 | M4 | M5 | M5 | M5 | M3 | M4 | M0 | M4 | M0 | M4 | M2 | M5 | M5 | M0 |
| 50 | M5 | M5 | M5 | M5 | M5 | M5 | M3 | M5 | M0 | M3 | M0 | M3 | M1 | M1 | M3 |
