# Supplementary material for: Assessment of brachial plexus and upper-extremity peripheral nerve injuries at the anatomical level using multimodal generative models
Source: Front Surg. 2026 Aug 13;13:1814943. doi: 10.3389/fsurg.2026.1814943 (PMC13518590; doi:10.3389/fsurg.2026.1814943)
Supplement: Supplementary file 2 [file Datasheet2.docx]

Supplementary Table 2 – Performance metrics, statistical calculations, binary scoring matrix, and response links

All analyses are based on the same 50 cases evaluated by four models. Binary coding: 1 = correct anatomical localization; 0 = incorrect localization.

# S1. Overall model accuracy

| **Model** | **Correct/total** | **Accuracy** | **Wilson 95% CI** |
| --- | --- | --- | --- |
| GPT-5 | 39/50 | 78.0% | 64.8–87.2% |
| Grok 4 | 26/50 | 52.0% | 38.5–65.2% |
| Gemini 2.5 Pro | 22/50 | 44.0% | 31.2–57.7% |
| Claude Opus 4.1 | 26/50 | 52.0% | 38.5–65.2% |

# S2. Cochran's Q omnibus test

Model totals Cj = [39, 26, 22, 26]; grand total T = 113; ΣCj² = 3357; ΣRi² = 359.

Q = (k−1)[kΣCj²−T²]/[kT−ΣRi²].

With k = 4 and n = 50: Q = 21.258065, df = 3, p = 0.00009305 (reported in the manuscript as p < 0.001).

# S3. Exact McNemar pairwise comparisons with Holm correction

| **Comparison** | **Both correct (a)** | **Model 1 only (b)** | **Model 2 only (c)** | **Both incorrect (d)** | **Matched-pair OR** | **Exact p** | **Holm-adjusted p** | **Interpretation** |
| --- | --- | --- | --- | --- | --- | --- | --- | --- |
| GPT-5 vs Grok 4 | 25 | 14 | 1 | 10 | 14.00 | 0.000977 | 0.004883 | Significant |
| GPT-5 vs Gemini 2.5 Pro | 20 | 19 | 2 | 9 | 9.50 | 0.000221 | 0.001328 | Significant |
| GPT-5 vs Claude Opus 4.1 | 24 | 15 | 2 | 9 | 7.50 | 0.002350 | 0.009399 | Significant |
| Grok 4 vs Gemini 2.5 Pro | 16 | 10 | 6 | 18 | 1.67 | 0.454498 | 1.000000 | Not significant |
| Grok 4 vs Claude Opus 4.1 | 20 | 6 | 6 | 18 | 1.00 | 1.000000 | 1.000000 | Not significant |
| Gemini 2.5 Pro vs Claude Opus 4.1 | 18 | 4 | 8 | 20 | 0.50 | 0.387695 | 1.000000 | Not significant |

Exact two-sided McNemar p values were calculated from the b+c discordant pairs using a binomial test under p = 0.5. Holm adjustment was applied across all six comparisons.

# S4. Category-specific descriptive accuracy

| **Category** | **Pooled correct/total** | **Pooled accuracy** | **Pooled Wilson 95% CI** | **GPT-5** | **Grok 4** | **Gemini 2.5 Pro** | **Claude Opus 4.1** |
| --- | --- | --- | --- | --- | --- | --- | --- |
| Roots | 22/40 | 55.0% | 39.8–69.3% | 9/10 (90%) | 5/10 (50%) | 3/10 (30%) | 5/10 (50%) |
| Trunks | 30/40 | 75.0% | 59.8–85.8% | 10/10 (100%) | 9/10 (90%) | 5/10 (50%) | 6/10 (60%) |
| Cords | 26/40 | 65.0% | 49.5–77.9% | 9/10 (90%) | 4/10 (40%) | 7/10 (70%) | 6/10 (60%) |
| Peripheral nerves | 27/40 | 67.5% | 52.0–79.9% | 7/10 (70%) | 6/10 (60%) | 6/10 (60%) | 8/10 (80%) |
| Combination injuries | 8/40 | 20.0% | 10.5–34.8% | 4/10 (40%) | 2/10 (20%) | 1/10 (10%) | 1/10 (10%) |

# S5. Individual-structure descriptive accuracy

| **Structure** | **Correct/total** | **Accuracy** | **Wilson 95% CI** |
| --- | --- | --- | --- |
| C5 | 19/32 | 59.4% | 42.3–74.5% |
| C6 | 21/40 | 52.5% | 37.5–67.1% |
| C7 | 11/24 | 45.8% | 27.9–64.9% |
| C8 | 11/16 | 68.8% | 44.4–85.8% |
| T1 | 11/16 | 68.8% | 44.4–85.8% |
| Upper trunk | 21/32 | 65.6% | 48.3–79.6% |
| Middle trunk | 14/32 | 43.8% | 28.2–60.7% |
| Lower trunk | 10/20 | 50.0% | 29.9–70.1% |
| Lateral cord | 12/20 | 60.0% | 38.7–78.1% |
| Posterior cord | 16/28 | 57.1% | 39.1–73.5% |
| Medial cord | 12/20 | 60.0% | 38.7–78.1% |
| Radial/PIN | 11/24 | 45.8% | 27.9–64.9% |
| Median nerve | 11/24 | 45.8% | 27.9–64.9% |
| Ulnar nerve | 3/20 | 15.0% | 5.2–36.0% |
| Axillary nerve | 4/4 | 100.0% | 51.0–100.0% |
| Musculocutaneous nerve | 4/4 | 100.0% | 51.0–100.0% |
| Suprascapular nerve | 7/8 | 87.5% | 52.9–97.8% |

# S6. Complete binary scoring matrix

| **Case** | **GPT-5** | **Grok 4** | **Gemini 2.5 Pro** | **Claude Opus 4.1** |
| --- | --- | --- | --- | --- |
| Case 1 | 1 | 1 | 1 | 1 |
| Case 2 | 1 | 1 | 0 | 1 |
| Case 3 | 0 | 0 | 0 | 1 |
| Case 4 | 1 | 0 | 0 | 0 |
| Case 5 | 1 | 0 | 0 | 0 |
| Case 6 | 1 | 1 | 0 | 0 |
| Case 7 | 1 | 0 | 0 | 0 |
| Case 8 | 1 | 0 | 0 | 0 |
| Case 9 | 1 | 1 | 1 | 1 |
| Case 10 | 1 | 1 | 1 | 1 |
| Case 11 | 1 | 1 | 1 | 1 |
| Case 12 | 1 | 1 | 1 | 1 |
| Case 13 | 1 | 1 | 1 | 1 |
| Case 14 | 1 | 0 | 0 | 0 |
| Case 15 | 1 | 1 | 0 | 0 |
| Case 16 | 1 | 1 | 0 | 0 |
| Case 17 | 1 | 1 | 1 | 1 |
| Case 18 | 1 | 1 | 0 | 0 |
| Case 19 | 1 | 1 | 0 | 1 |
| Case 20 | 1 | 1 | 1 | 1 |
| Case 21 | 1 | 1 | 1 | 1 |
| Case 22 | 0 | 0 | 1 | 0 |
| Case 23 | 1 | 1 | 1 | 1 |
| Case 24 | 1 | 0 | 1 | 1 |
| Case 25 | 1 | 0 | 0 | 0 |
| Case 26 | 1 | 0 | 1 | 0 |
| Case 27 | 1 | 0 | 0 | 1 |
| Case 28 | 1 | 1 | 1 | 1 |
| Case 29 | 1 | 1 | 1 | 1 |
| Case 30 | 1 | 0 | 0 | 0 |
| Case 31 | 1 | 0 | 1 | 1 |
| Case 32 | 1 | 1 | 0 | 1 |
| Case 33 | 1 | 1 | 1 | 1 |
| Case 34 | 1 | 1 | 1 | 1 |
| Case 35 | 0 | 0 | 0 | 0 |
| Case 36 | 0 | 0 | 0 | 1 |
| Case 37 | 1 | 1 | 1 | 1 |
| Case 38 | 1 | 1 | 1 | 1 |
| Case 39 | 1 | 1 | 0 | 1 |
| Case 40 | 0 | 0 | 1 | 0 |
| Case 41 | 1 | 0 | 0 | 1 |
| Case 42 | 1 | 1 | 0 | 0 |
| Case 43 | 1 | 0 | 1 | 0 |
| Case 44 | 0 | 1 | 0 | 0 |
| Case 45 | 0 | 0 | 0 | 0 |
| Case 46 | 0 | 0 | 0 | 0 |
| Case 47 | 0 | 0 | 0 | 0 |
| Case 48 | 0 | 0 | 0 | 0 |
| Case 49 | 0 | 0 | 0 | 0 |
| Case 50 | 1 | 0 | 0 | 0 |

# S7. Source links to model responses

| **Batch** | **GPT-5** | **Grok 4** | **Gemini 2.5 Pro** | **Claude Opus 4.1** |
| --- | --- | --- | --- | --- |
| Batch 1 | [Open response](https://chatgpt.com/share/68a0482b-31ac-8006-8ff8-c4468c1198e1) | [Open response](https://grok.com/share/c2hhcmQtMw%3D%3D_0588f135-cbc8-4155-acfe-f40400f63535) | [Open response](https://g.co/gemini/share/6d822241668a) | [Open response](https://claude.ai/share/52a37047-21b4-43f7-81cf-1001cea5da63) |
| Batch 2 | [Open response](https://chatgpt.com/share/68a050c8-49fc-8006-bbb7-c665511498bb) | [Open response](https://grok.com/share/c2hhcmQtMw%3D%3D_7fc34717-6453-4828-83f5-471a1f7b1492) | [Open response](https://g.co/gemini/share/2bcd44f328a4) | [Open response](https://claude.ai/share/61226bd3-f178-4713-88de-ef79fcd5c319) |
| Batch 3 | [Open response](https://chatgpt.com/share/68a05e28-46e8-8006-be16-694d6fff62d4) | [Open response](https://grok.com/share/c2hhcmQtMw%3D%3D_e9268809-126b-4843-8d97-5fca32e3e9ea) | [Open response](https://g.co/gemini/share/862512d62bdc) | [Open response](https://claude.ai/share/f2336940-18bf-4ffe-bf0c-a706ab4f01d3) |
| Batch 4 | [Open response](https://chatgpt.com/share/68a08dd5-582c-8006-ab97-56ddeb8a1e51) | [Open response](https://grok.com/share/c2hhcmQtMw%3D%3D_7af5635e-9e04-4880-8de5-0b5e7aff8783) | [Open response](https://g.co/gemini/share/18de568361f4) | [Open response](https://claude.ai/share/d3706241-c803-47b3-8290-89cb54d0b76e) |
| Batch 5 | [Open response](https://chatgpt.com/share/68a095c6-fabc-8006-adea-f58fa9d2c9fc) | [Open response](https://grok.com/share/c2hhcmQtMw%3D%3D_e5131921-925d-467d-811e-10e290d82226) | [Open response](https://g.co/gemini/share/7dcbf5806721) | [Open response](https://claude.ai/share/7c53d465-20a2-48d0-8170-09c75d91ee87) |

The five batches correspond to the five predefined case groups (10 cases per batch). Hyperlinks open the shared model responses; accessibility depends on the continued availability and sharing settings of the respective provider.
